# A generalist vector-transmitted parasite exhibits population genetic structure among host genera

**DOI:** 10.1017/S0031182024001641

**Published:** 2025-01

**Authors:** Vincenzo A. Ellis, Mélanie Duc, Arif Ciloglu, Olof Hellgren, Staffan Bensch

**Affiliations:** 1 Department of Entomology and Wildlife Ecology, University of Delaware, Newark, DE, USA; 2 P. B. Šivickis Laboratory of Parasitology, Nature Research Centre, Vilnius, Lithuania; 3 Department of Parasitology, Faculty of Veterinary Medicine, Erciyes University, Kayseri, Türkiye; 4 Vectors and Vector-Borne Diseases Implementation and Research Center, Erciyes University, Kayseri, Türkiye; 5 Department of Biology, Lund University, Lund, Sweden

**Keywords:** avian haemosporidians, host specificity, parasite evolution, pathogen, population genomics

## Abstract

Generalist parasites experience selective pressures from the various host species they infect. However, it is unclear if parasite transmission among host species precludes the establishment of host-specific adaptations and population genetic structure. We assessed the population genetic structure of the vector-transmitted avian haemosporidian parasite *Haemoproteus majoris* (lineage WW2; *n* = 34 infections) in a single site in southern Sweden among 10 of its host species. The 2 best-sampled host genera were *Phylloscopus* (2 species, *n* = 15 infections) and *Sylvia* (4 species, *n* = 15). We designed a sequence capture protocol to isolate 1.13 Mbp (*ca.* 5%) of the parasite genome and identified 1399 variable sites among the sequenced infections. In a principal components analysis, infections of *Phylloscopus* and *Sylvia* species mostly separated along the first 2 principal components. Sites with the highest *F*_ST_ values between the genera were found in genes that have mostly not been implicated in infection pathways, but several sites code for amino acid changes. An analysis of molecular variance confirmed significant variation among host genera, but not among host species within genera. The distribution of Tajima’s *D* among sequenced loci was negatively skewed, plausibly reflecting a history of bottleneck followed by population expansion. Tajima’s *D* was lower in infections of *Phylloscopus* than *Sylvia*, plausibly because WW2 began infecting *Phylloscopus* hosts after it was already a parasite of *Sylvia* hosts. Our results provide evidence of vector-transmitted parasite population differentiation among host species in a single location. Future work should focus on identifying the mechanisms underlying this genetic population structure.

## Graphical abstract

**Figure:**
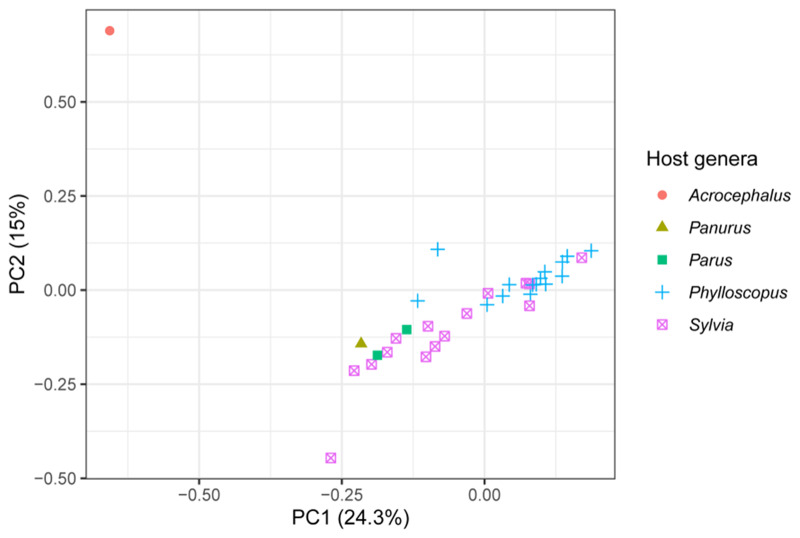

## Introduction

Host specificity is a fundamental parasite phenotype and is hypothesized to influence infectious disease emergence (Woolhouse and Gowtage-Sequeria, 2005). Parasites range from specialists that infect 1 or a few (often closely related) host species to generalists that infect many host species across varying degrees of taxonomic levels. Theoretical and empirical investigations have largely focused on testing whether host specificity is determined by evolutionary trade-offs between generalist and specialist strategies (Visher and Boots, 2020). One type of trade-off that would favour the evolution of specialists over generalists involves antagonistic pleiotropy, wherein an allele that increases a parasite’s fitness in 1 host, decreases its fitness in another host (Visher and Boots, 2020). Under a scenario of antagonistic pleiotropy, one might expect a single population of a generalist to evolve into distinct genetic populations that specialize on each of the generalist’s host species. The establishment of such genetic population structure might then lead to sympatric speciation of the generalist into various specialists. In the case of vector-transmitted parasites, generalists might also establish population structure because of vector biting preference. While there is evidence that generalists beget specialists over evolutionary time (Loiseau et al., 2012), closely related generalists have been identified in some groups (Mouillot et al., 2006; Ellis and Bensch, 2018) suggesting that generalists may not always evolve into specialists.

Few studies have investigated the population genetic structure of generalist parasites among their host species in the wild. In a study of a generalist nematode parasite of sympatric ungulates, Archie and Ezenwa (2011) found no population genetic differentiation in the nematode among its host species. Conversely, the seabird tick *Ixodes uriae* shows host-specific genetic population structure across its distribution (McCoy et al., 2005). Sympatric speciation and changes in host specificity, whether or not preceded by the establishment of genetic population structure, can happen rapidly. For example, the ancestor of the 2 mosquito-vectored parasites *Plasmodium falciparum* and *P. praefalciparum* appears to have acquired a gene through introgression (Otto et al., 2018) that allowed it to infect both gorillas and humans. This was followed by a relatively small number of mutations restricting *P. falciparum* to humans (Galaway et al., 2019) and its sister species (*P. praefalciparum*) to gorillas; the 2 parasites appear to have diverged about 50 kya (Otto et al., 2018).

Avian haemosporidians (often called avian malaria and related parasites) are nearly globally distributed dipteran-vectored protozoan parasites (Valkiūnas, 2005). Genetic lineages of avian haemosporidians are defined by a barcoding region of the parasite cytochrome b gene (Bensch et al., 2009) and exhibit great variation in host specificity (Ellis and Bensch, 2018). The avian haemosporidian species *Haemoproteus majoris* is composed of several genetic lineages including both generalists and specialists (Nilsson et al., 2016). In southern Sweden, the *H. majoris* lineage WW2 frequently infects warblers from the genera *Phylloscopus* and *Sylvia* and, to a lesser degree, host species of other genera (Huang et al., 2018b; Ellis et al., 2020). The prevalence of WW2 is positively correlated with parasitemia among host species, and WW2 has higher prevalence and parasitemia in *Phylloscopus* and *Sylvia* warblers than in other host species (Huang et al., 2018b). While avian haemosporidian vectors have often been shown to be generalist biters (e.g. Medeiros et al., 2013), the vectors of WW2 in southern Sweden are unknown. However, at a similar latitude in Lithuania, the biting midges *Culicoides pictipennis, C. segnis* and *C. kibunensis* have been put forward as potential vectors of *H. majoris* (Žiegytė et al., 2022). All 3 species have been found in southern Sweden where WW2 has been studied (unpublished data). An initial investigation of 4 parasite nuclear genes showed no genetic population structure in WW2 among its host species and little genetic variation overall (Nilsson et al., 2016). However, it remains unclear if those results hold across a broader sample of the parasite genome.

Sequencing avian haemosporidian genomes is challenging because of the high abundance of host DNA in a sample of infected host blood (Videvall, 2019). Several techniques have been used to overcome this obstacle including transcriptome sequencing (Videvall et al., 2017; Galen et al., 2020) and genomic sequence capture (Huang et al., 2018a; Barrow et al., 2019; Ellis et al., 2022). While transcriptome sequencing requires preserving RNA, sequence capture can be performed on previously collected DNA, making it particularly useful for retrospective analyses.

Huang et al. (2018b) designed sequence capture probes based on the genome of *H. tartakovskyi* and used those probes to capture and sequence part of the genome of WW2. We used the WW2 sequences from Huang et al. (2018b) to design new sequence capture probes targeting 1.13 Mbp of WW2 genes, *ca.* 5% of the parasite genome (Bensch et al., 2016). We then performed sequence capture on WW2 infections identified in wild birds captured in southern Sweden (Huang et al., 2018b; Ellis et al., 2020). We used the resulting genomic data to test whether WW2 showed population genetic structure among its host species and genera and to identify the genes, if any, that show the greatest evidence of host-specific structure.

## Materials and methods

### Sample identification

In a previous study, we quantified the parasitemia of WW2 in avian DNA samples collected at lake Krankesjön (55°41′N, 13°26′E) in southern Sweden between 2013 and 2016 (Huang et al., 2018b). We identified 48 samples with relatively high parasitemia values and representing a diversity of host species for use in this study (host species information presented in Supplementary Table S1). Of those 48 infections, 34 passed our variant filtering (see *Bioinformatic processing* subsection) and were analysed further.

### Sequence capture

Sequence capture uses biotinylated RNA probes to capture genetic sequences of interest. As a basis for probe design, we identified 1497 regions from nuclear exons (hereafter ‘loci’) from WW2 sequenced in a previous sequence capture study that used probes targeting *H. tartakovskyi* (GenBank BioProject accession number PRJNA448510; Huang et al., 2018b). To those sequences, we added the nearly complete WW2 mitochondrial genome (5850 bp) amplified from a sample that we used for sequence capture (sample H11; Supplementary Table S1). The mitochondrial genome (GenBank accession number: PP797599) was PCR amplified and sequenced following the protocol outlined in Ciloglu et al. (2020). Finally, we added sequences of the caseinolytic protease C (clpC) gene of the apicoplast genome, based on the available sequences of 2 congeneric parasites, *H. magnus* (GenBank accession number: EU254647.1) and *H. passeris* (EU254653.1). We put all sequences together into a single FASTA file and provided this to TATAA Biocenter, a company that works with Arbor Biosciences to distribute myBaits probes. Arbor Biosciences removed 32 of the nuclear loci from the file, leaving 1468 targeted loci (1465 nuclear exon regions, the mitochondrial genome, and the 2 congeneric clpC sequences). The sequence names and corresponding gene identification numbers targeted from the *H. tartakovskyi* genome (Bensch et al., 2016) are provided in Supplementary Table S2. We also included the latest gene annotation and descriptions in Supplementary Table S2 using the *H. tartakovskyi* annotation in version 67 of the PlasmoDB website (https://plasmodb.org/; Alvarez-Jarreta et al., 2024); several of the genes from the original study (Bensch et al., 2016) did not map or are not annotated as genes in the latest PlasmoDB version. For genes of interest with high *F*_ST_ values among infections in the host genera *Phylloscopus* and *Sylvia*, we provide original gene annotations and descriptions (Bensch et al., 2016) if they differ from the latest annotation. We found the latest gene annotation information by mapping the originally identified genes of *H. tartakovskyi* (Bensch et al., 2016) to the current PlasmoDB reference genome with BWA MEM v.0.7.17 (Li, 2013), converting the resulting SAM file to bam format with Samtools v.1.19.2 view (Danecek et al., 2021) and then to bed format with bedtools v.2.30.0 bamtobed (Quinlan and Hall, 2010) and finally extracting the gene information with bedtools intersect and further processing in R v.4.2.2 (R Core Team, 2022) with tidyverse packages (Wickham et al., 2019). The FASTA file of targeted loci is provided in Supplementary File 1. Arbor Biosciences then provided us with a FASTA file of 16 850 probes, each 120 bp in length, covering our targeted sequences using 2× tiling (Supplementary File 2).

We received the synthesized probes as part of the myBaits hybridization capture for targeted NGS kit. We used this in conjunction with the Swift Biosciences Accel-NGS 2S Hyb DNA Library Kit and KAPA HiFi HotStart ReadyMix PCR Kit to perform sequence capture on our samples following the manufacturer’s instructions. Briefly, we sheared the 48 DNA samples using an M220 Focused-ultrasonicator (Covaris, MA, USA) and then built indexed Illumina libraries from all samples. We next made 6 pools, each made up of 8 libraries at equimolar concentrations, and we performed the sequence capture on each pool separately. We sequenced the captured DNA on an Illumina MiSeq (paired-end 300bp) at the Lund University DNA Sequencing Facility. All raw sequence data are available on GenBank (accession number: PRJNA1111424).

### Bioinformatic processing

We assessed the quality of raw sequencing data with FastQC v.0.11.9 (https://www.bioinformatics.babraham.ac.uk/projects/fastqc/). We then removed sequencing adapters and performed quality trimming using Trim Galore v.0.6.6 (https://github.com/FelixKrueger/TrimGalore) with the following flags: −−quality 20, −−length 50. We next mapped the paired reads to the target sequences (Supplementary File 1) using BWA MEM v.0.7.17 (Li, 2013). We used SAMtools v.1.19.2 (Danecek et al., 2021) to generate mapping statistics (SAMtools stats) and convert the resulting mapped read files to sorted bam format (SAMtools view and sort). Next, we marked duplicates in the bam files with Picard v.3.1.1 (https://github.com/broadinstitute/picard) MarkDuplicates and added read group information to the bam files with Picard AddOrReplaceReadGroups. We then indexed the bam files with SAMtools index and called variants with GATK v.4.3.0 (Poplin et al., 2017; Auwera and O’Connor, 2020) HaplotypeCaller with the flags -ploidy 1 (to designate haploid samples) and -ERC GVCF; we prepared the reference file (Supplementary File 1) for HaplotypeCaller with SAMtools faidx and Picard CreateSequenceDictionary. We combined the resulting gzipped GVCF files with GATK CombineGVCFs followed by GATK GenotypeGVCFs. We ran most of these steps with the GNU Parallel program v.20230122 (Tang, 2023). After generating a single gzipped VCF file for all sequenced samples, we examined variant statistics in VCFtools v.0.1.16.4 (Danecek et al., 2011) and BCFtools v.1.17 (Danecek et al., 2021) and R with packages in the tidyverse. We used BCFtools to examine variants in the barcoding region of the mitochondrial genome (mtDNAgenome_CP59298, positions 4447–4925); samples with variants in that region were assumed to be mixed lineage infections and were removed from the VCF file and not analysed (all variants in that region had a minimum quality score of 30). Hard filtering VCF files is a balance between retaining samples and variable sites and removing poor quality variant calls. We filtered the VCF file with VCFtools using the following flags: −−max-missing 0.5 −−minQ 30 −−min-meanDP 4 −−max-meanDP 250 −−minDP 4 −−maxDP 250. Finally, sites in the VCF file with missing data were converted back to haploid format (from ‘./.’ to ‘.’) using BCFtools fixploidy. The filtering left us with 34 samples and 1399 variable sites (1017 single nucleotide polymorphisms [SNPs] and 411 insertions/deletions [indels]; some variable sites include both SNPs and indels) among the samples in our final VCF.

### Statistical analysis

We conducted a principal components analysis (PCA) in PLINK v.1.90 (Purcell et al., 2007). We started by removing variants in linkage disequilibrium calculated using 5 kb windows, a 10 bp step size, and an *r^2^* cut-off value of 0.20. Next, we ran the PCA on the reduced dataset and plotted the samples in multivariate space with R and packages in the tidyverse. We calculated the Weir and Cockerham weighted *F*_ST_ and *F*_ST_ per variable site among host species and genera using VCFtools (−−weir-fst-pop). However, we downloaded a forked version of VCFtools (https://github.com/jydu/vcftools.git) that was modified to allow haploid variants, which we did with the flag – haploid. For several variable sites with high *F*_ST_ between the 2 best sampled genera (*Phylloscopus* and *Sylvia*), we conducted a BLAST search of the sequenced target region (i.e. gene) on GenBank to make sure the pattern was not caused by host reads mapping to those sites. Avian genes were not among the top BLAST hits for any of the sites, and we therefore conclude that the results reflect true parasite genetic differences (results not shown). We were also interested in classifying variants as synonymous or non-synonymous. To do this we extracted consensus sequences using BCFtools consensus and organized the output into multiple sequence alignments (MSAs) using custom awk code. We then aligned the MSAs of loci with high *F*_ST_ between the 2 best sampled genera (*Phylloscopus* and *Sylvia*) with their corresponding genes from the *H. tartakovskyi* assembly in version 67 of the PlasmoDB website using Geneious v.11.1.5 (http://www.geneious.com/). Next, we extracted exon regions only from the MSAs and imported them into R as FASTA files with the R package ape v.5.7.1 (Paradis and Schliep, 2019). We then used the R package pegas v.1.3 (Paradis, 2010) to identify the haplotypes (function haplotype()) within each MSA and count the number of nucleotide (function dist.dna (model = ‘N’)) and amino acid (functions trans(code = 1) and dist.aa()) differences between the haplotypes (alignments were confirmed to be in the correct reading frame based using the corresponding reference gene). We calculated nucleotide diversity (π; −−window-pi 1000) and Tajima’s *D* (−−Tajima*D* 1000) over 1 kb windows using the forked version of VCFtools for all infections and infections of *Phylloscopus* and *Sylvia* hosts separately; we compared π and Tajima’s *D* between infections of the 2 host genera using paired *t*-tests (paired because the same genomic windows were compared) with the R function t.test() (paired, non-parametric Wilcoxon tests gave similar results and are not reported). Finally, we read our VCF file into R with the package vcfR v.1.15.0 (Knaus and Grünwald, 2017) and conducted an analysis of molecular variance (AMOVA) in the package poppr v.2.9.6 (Kamvar et al., 2014) with the function poppr.amova() using the hierarchical strata of host genus and host species within genus as explanatory variables, the ade4 implementation of AMOVA, and the argument missing = ‘ignore’. We tested the significance of the AMOVA results with the randtest() function with 999 randomizations (nrepet = 999) in the package ade4 v.1.7-22 (Chessel et al., 2004; Dray and Dufour, 2007; Dray et al., 2007; Bougeard and Dray, 2018; Thioulouse et al., 2018). We visualized the phylogenetic relationships among host species by making a consensus phylogeny of 100 trees from the posterior distribution of the analysis from birdtree.org (Jetz et al., 2012); we did this with the functions consensus.edges() in the R package phytools v.2.0-3 (Revell, 2012) and functions in ape.

## Results

**Figure 1.**
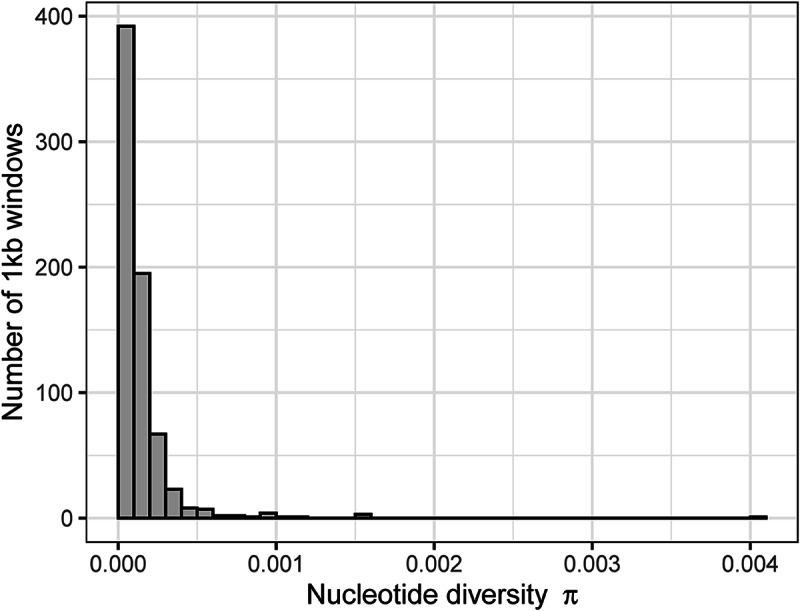
Distribution of nucleotide diversity (π) in the parasite lineage WW2 calculated over 1 kb windows.

**Figure 2.**
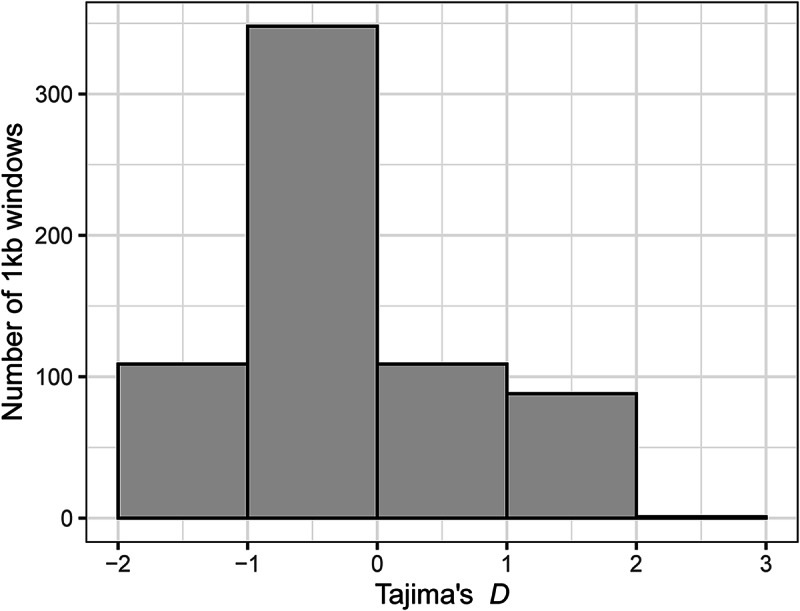
Distribution of Tajima’s *D* values in the parasite lineage WW2 calculated over 1 kb windows.

We identified 14 infections with variant calls in the barcoding region of the mitochondrial genome. Because such variation in that region is suggestive of mixed lineage infections (in the avian haemosporidian system, 1 nucleotide difference in the barcoding region of cytochrome b is considered a unique lineage; Bensch et al., 2009), we removed those samples from the analysis. After additional quality filtering, our final VCF file included 34 samples (i.e. infections) and 1399 variable sites (Supplementary File 3) representing infections from 10 host species and 5 host genera (Table 1; Supplementary Fig. S1). Nucleotide diversity (π) ranged from 2.85 × 10^−5^ to 4.04 × 10^−3^ (mean = 1.38 × 10^−4^ ± 2.21 × 10^−4^ S.D.; Figure 1). Tajima’s *D* ranged from −1.84 to 2.16 (−0.29 ± 0.883; Figure 2). While π was higher in infections of *Phylloscopus* hosts than in infections of *Sylvia* hosts (Supplementary Fig. S2; *t* = 14.03, D.F. = 338, *P* < 0.001), Tajima’s *D* was lower in infections of *Phylloscopus* hosts than in infections of *Sylvia* hosts (Supplementary Fig. S3; *t* = −7.52, D.F. = 292, *P* < 0.001).

**Table 1.** Number of infections (*N*) of the lineage WW2, parasite *H. majoris*, analysed by host species after variant filtering and removing mixed parasite lineage infections

Host species	*N*
*Phylloscopus trochilus*	8
*Phylloscopus collybita*	7
*Sylvia communis*	7
*Sylvia atricapilla*	4
*Sylvia curruca*	3
*Sylvia borin*	1
*Acrocephalus scirpaceus*	1
*Panurus biarmicus*	1
*Parus ater*	1
*Parus palustris*	1

**Figure 3.**
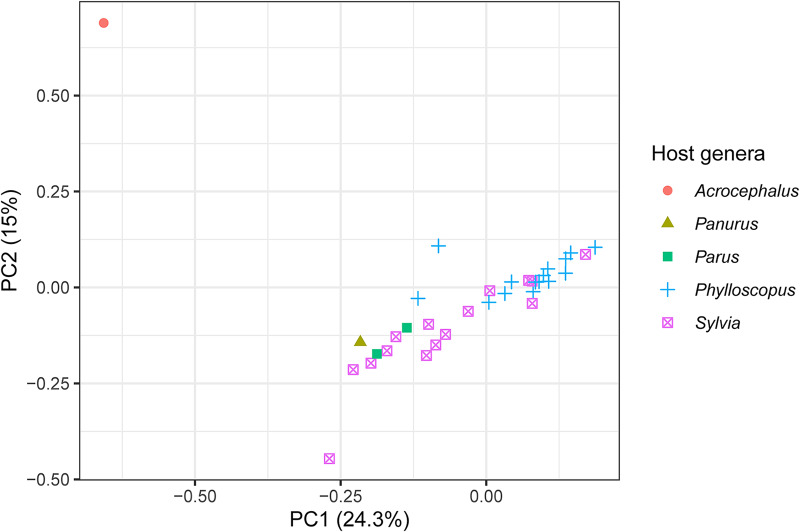
The first 2 principal components from a PCA of genetic variation among isolates of the parasite lineage WW2 with host genus highlighted. Infections from the 2 best sampled host genera, *Phylloscopus* and *Sylvia*, mostly separate along the first 2 principal components. The variation in the genetic data explained by each principal component is presented in the axis labels. The PCA used 20 axes in total.

The 2 best sampled host genera were *Phylloscopus* and *Sylvia* and they mostly separated in the multivariate space of a PCA along the first 2 principal component axes, representing 24.3% and 15% of the variation in the genetic data from the PCA’s 20 axes, respectively (Figure 3). The lone infection of *Acrocephalus scirpaceus* also separated from the other infections (Figure 3). The loadings of the variable sites on the first 2 principal components are presented in Supplementary Table S3.

The weighted *F*_ST_ among all genera with more than 1 host species in the data (*Phylloscopus, Sylvia* and *Parus*) was 0.084 and among species with more than 1 infection (Table 1) was 0.065. The weighted *F*_ST_ between infections of the host genera *Phylloscopus* and *Sylvia* was 0.023. When comparing those 2 genera, there were 524 variable sites with *F*_ST_ > 0. We calculated the 95^th^ (0.351) and 99^th^ (0.655) percentiles of the 524 positive *F*_ST_ values. There were 27 *F*_ST_ values higher than the 95^th^ percentile, including 6 higher than the 99^th^ percentile (Table 2). Only 1 of these high *F*_ST_ values appeared to be related to infection pathways (HtGene0230, merozoite capping protein 1; Table 2). We were able to calculate the number of synonymous and non-synonymous substitutions for 6 genes (annotated as protein coding genes in the reference genome) with high *F*_ST_ values and found both synonymous and non-synonymous mutations (Table 3). The remaining genes could not be classified for various reasons (e.g. only 1 haplotype in the exon region of the reference, challenges with identifying the correct reading frame after alignment).

**Table 2.** Variable sites of the parasite lineage WW2 with F_ST_ values between infections in the hosts of the genera *Phylloscopus* and *Sylvia*. Only variable sites with F_ST_ values greater than the 95^th^ percentile of all positive F_ST_ values are shown and sites with F_ST_ values greater than the 99^th^ percentile (column 99%) are indicated as ‘outliers’. Target refers to the targeted sequence that the sequence capture probes were designed to capture; POS is the position on that sequence that was variable; HtGene is the name of the gene in the original published assembly of the reference *H. tartakovskyi* genome; RefGene is the corresponding gene name in the latest assembly; type indicates whether the region is characterized as a protein coding gene or pseudogene in the latest assembly; gene function is the annotated description of the gene in the latest assembly, and the note ‘original annotation’: refers to the annotated description from the original assembly; both the latest and the original assembly descriptions are provided if the gene annotation differs between the assemblies. Missing RefGene and type values indicate that the gene from the original assembly is not annotated in the latest assembly. The F_ST_ values for the variable sites are also shown

Target	POS	*F*_ST_	99%	HtGene	RefGene	Type	Gene function
seq73__HtGene0129	161	0.693	Outlier	HtGene0129			Original annotation: Putative uncharacterized protein OS = Plasmodium
seq75__HtGene0131	1147	0.351		HtGene0131	Htart_000018100	Pseudogene	DNA primase large subunit putative
seq93__HtGene0183	1003	0.678	Outlier	HtGene0183	Htart_000022800	Protein coding gene	Metacaspase-like protein; Original annotation: Putative uncharacterized protein OS = Plasmodium
seq114__HtGene0230	517	0.529		HtGene0230			Original annotation: Merozoite capping protein 1, putative
seq120__HtGene0243	950	0.365		HtGene0243	Htart_000029900	Protein coding gene	Methionine aminopeptidase 1b putative
seq255__HtGene0476	126	0.408		HtGene0476	Htart_000054900	Protein coding gene	TFIIH basal transcription factor complex helicase XPD subunit; Original annotation: DNA excision-repair helicase, putative
seq258__HtGene0481	71	0.382		HtGene0481	Htart_000055400	Protein coding gene	V-type proton ATPase subunit E putative
seq608__HtGene1356	382	0.360		HtGene1356	Htart_000141500	Pseudogene	Elongation factor 2
seq664__HtGene1491	78	0.680	Outlier	HtGene1491	Htart_000154400	Pseudogene	Pre-mRNA-splicing factor 38A putative; Original annotation: Putative uncharacterized protein OS = Plasmodium
seq665__HtGene1491	483	0.703	Outlier	HtGene1491	Htart_000154400	Pseudogene	Pre-mRNA-splicing factor 38A putative; Original annotation: Putative uncharacterized protein OS = Plasmodium
seq790__HtGene1778	212	0.367		HtGene1778			Original annotation: Apicoplast ribosomal protein L10, putative
seq927__HtGene2105	261	0.409		HtGene2105	Htart_000212900	Protein coding gene	LCCL domain-containing protein
seq956__HtGene2156	339	0.351		HtGene2156	Htart_000217300	Protein coding gene	Exoribonuclease putative
seq1151__HtGene2635	473	0.648		HtGene2635	Htart_000261100	Protein coding gene	Vacuolar proton translocating ATPase subunit A putative
seq1168__HtGene2682	72	0.463		HtGene2682	Htart_000266200	Pseudogene	Myosin C
seq1187__HtGene2704	433	0.462		HtGene2704	Htart_000268400	Protein coding gene	CCR4-associated factor 1; Original annotation: Putative uncharacterized protein (Fragment)
seq1234__HtGene2808	407	0.351		HtGene2808			Original annotation: RNA binding protein, putative OS = Plasmodium
seq1413__HtGene3214	1546	0.608		HtGene3214			Original annotation: Putative uncharacterized protein OS = Medicago
seq1487__HtGene3342	99	0.363		HtGene3342	Htart_000331000	Pseudogene	DNA gyrase subunit A
seq1487__HtGene3342	100	0.363		HtGene3342	Htart_000331000	Pseudogene	DNA gyrase subunit A
seq1526__HtGene3475	517	0.657	Outlier	HtGene3475	Htart_000342800	Protein coding gene	Mitosis protein dim1 putative
seq1533__HtGene3504	31	0.462		HtGene3504	Htart_000345400	Protein coding gene	40S ribosomal protein S7 putative
seq1664__HtGene3763	45	0.682	Outlier	HtGene3763	Htart_000368400	Protein coding gene	FeS cluster assembly protein SufD putative
seq1703__HtGene3892	36	0.370		HtGene3892	Htart_000381900	Protein coding gene	Zinc finger protein putative; Original annotation: Putative uncharacterized protein OS = Plasmodium
seq1703__HtGene3892	39	0.370		HtGene3892	Htart_000381900	Protein coding gene	Zinc finger protein putative; Original annotation: Putative uncharacterized protein OS = Plasmodium
seq1704__HtGene3893	1435	0.351		HtGene3893	Htart_000382000	Protein coding gene	Heat shock protein 90 putative
seq1718__HtGene3926	609	0.351		HtGene3926	Htart_000384900	Pseudogene	6-phosphofructokinase

**Table 3.** Target loci (target) with high F_ST_ values among *Phylloscopus* and *Sylvia* hosts (Table 2) were aligned with exons in the corresponding protein coding genes in the reference *H. tartakovskyi* assembly (RefGene); we were able to determine nucleotide substitution types (synonymous or non-synonymous) among haplotypes of 6 genes. We present the number of haplotypes per gene, the haplotype sequence length, mean and maximum number of nucleotide substitutions among haplotypes, and the corresponding mean and maximum number of amino acid substitutions in the translated haplotype sequences

Target	RefGene	Sequence length (bp)	Number of haplotypes	Nucleotide substitutions (mean/max)	Amino acid substitutions (mean/max)	Nucleotide substitution types
seq93__HtGene0183	Htart_000022800	1819	4	(1/1)	(0/0)	Synonymous
seq120__HtGene0243	Htart_000029900	1054	5	(22.9/52)	(9.4/19)	Synonymous and non-synonymous
seq927__HtGene2105	Htart_000212900	1188	3	(1.33/2)	(1.33/2)	Non-synonymous
seq1151__HtGene2635	Htart_000261100	549	2	(1/1)	(1/1)	Non-synonymous
seq1187__HtGene2704	Htart_000268400	893	5	(2/3)	(1.6/3)	Synonymous and non-synonymous
seq1664__HtGene3763	Htart_000368400	1569	3	(1.33/2)	(0.66/1)	Synonymous and non-synonymous

An AMOVA revealed significantly more variation than expected by chance among infections in different host genera (43.25% of the variance, *P* = 0.014) and less variation among infections of the same host species than expected by chance (52.32%, *P* = 0.001; Table 4).

**Table 4.** Results of analysis of molecular variance (AMOVA). the sources of variation were infections among host genera (‘host genera’), infections among host species within host genera (‘host species within genera’), and infections in host species (‘infections within host species’). Degrees of freedom (DF), sum of square deviations (SSD), mean square deviations (MSD), variance and its percent of the total variance; phi is the population differentiation statistic. The *p* value for each source of variance from a randomization test is also presented. Infections differed among host genera more than expected by chance and infections within host species differed less than expected by chance

	D.F.	SSD	MSD	Variance	Percent variance	Phi	*P*
Host genera	4	11778.69	2944.67	445.50	43.25	0.433	0.014
Host species within genera	5	3536.83	707.37	45.60	4.43	0.078	0.235
Infections within host species	24	12934.66	538.94	538.94	52.32	0.477	0.001

## Discussion

Using population genomic data from a vector transmitted generalist parasite, we found population structure among infections of different host genera, but not among infections of congeneric host species (Table 4). Infections of the 2 best sampled host genera, *Phylloscopus* and *Sylvia*, mostly separated in multivariate space of a PCA (Figure 3). The population structure was associated with genes that were largely unrelated to infection pathways (Table 2). However, 5 of 6 genes with high *F*_ST_ values between *Phylloscopus* and *Sylvia* hosts included non-synonymous mutations (Table 3). Lack of sufficient variation among the infections precluded more direct tests of selection (e.g. McDonald and Kreitman, 1991). Nevertheless, the large proportion of non-synonymous mutations is suggestive of selection. We therefore cannot distinguish between vector-mediated parasite isolation and host-mediated selection in causing the population genetic structure we observed. Nucleotide diversity (π; Figure 1) and, to a lesser extent, Tajima’s *D* (Figure 2) were negatively skewed. The latter is consistent with a demographic history of historical bottleneck in the parasite followed by population growth. We also identified several mixed lineage infections, which we did not analyse. However, future long-read sequencing may allow for analysis of such mixed lineage infections.

Parasite population structure caused by host-specific selection would plausibly be reflected in high *F*_ST_ values in genes involved in infection pathways, particularly genes that code for proteins that interact with host cell proteins. This may also be true of population structure among host genera since aspects of the host immune system are evolutionary conserved (O’Connor et al., 2016). Only gene HtGene0230 had a high *F*_ST_ among host genera and was involved in infection pathways (Table 2). In the original annotation of the *H. tartakovskyi* reference genome (Bensch et al., 2016), this gene was annotated as merozoite capping protein 1 (MCP1). MCP1 may be involved in the attachment of the merozoite to host red blood cells (Klotz et al., 1989). However, in the latest annotation of the *H. tartakovskyi* genome, the sequence is no longer annotated as a gene (Table 2). Despite the paucity of infection pathway genes contributing to WW2’s population structure among *Phylloscopus* and *Sylvia* hosts (Table 2), 5 of 6 investigated genes included non-synonymous nucleotide substitutions separating their haplotypes (Table 3). Parasites may also experience selection in genes in response to different host physiologies, and those genes may not necessarily be related to infection pathways. Another possibility is that genes with high *F*_ST_ among host genera are linked to infection related genes that we did not sequence. Besides host-specific selection, vectors of WW2 may have different preferences for bird genera; we cannot distinguish between these 2 possibilities with our data. Furthermore, host selection and vector preference may be operating simultaneously. Our results emphasize the need to better study vectors in this system and to characterize more genes in WW2’s genome. Characterizing parasite gene transcription during infection (Videvall et al., 2017) and among host species (in the case of generalists) remains an important endeavour (Garcia-Longoria et al., 2020). Furthermore, we sequenced relatively high intensity infections and it is unclear if lower intensity infections would show the same patterns.

Tajima (1989) examined the relationship between the number of segregating sites and the average number of pairwise differences between sequences of individuals in a neutrally evolving population. The *D* statistic was described as the difference between the pairwise nucleotide differences and the number of segregating sites (the latter scaled by sample size) divided by the standard deviation of that difference (Tajima, 1989). Negative values of *D* suggest more rare alleles than expected under a neutrally evolving population. A large negative *D* might result from a gene under selection, but here we found negative values between −1 and 0 of *D* across many of the genes surveyed (Figure 2). This result would appear consistent with a historical bottleneck followed by population expansion, since a growing population could produce an excess of new mutations (Hartl, 2020). While it is unclear what could have caused this, one can speculate. The closest relative to WW2 and the other *H. majoris* lineages might be a specialist of the host *Sylvia borin* (e.g. SYBOR15 was used as the closest relative of *H. majoris* in an analysis by Nilsson et al., 2016). Such a switch from specialist to generalist may have caused a bottleneck, followed by rapid population growth as the parasite began infecting additional host species. Indeed, Tajima’s *D* values were lower in infections of *Phylloscopus* hosts than in infections of *Sylvia* hosts (Supplementary Fig. S3) as might be expected if *Phylloscopus* hosts are more recent hosts of WW2. Testing for a relationship between evolutionary shifts in host specificity and parasite population genetics awaits better supported parasite phylogenies (perhaps through mitochondrial genome sequencing; Pacheco et al., 2018; Ciloglu et al., 2020) and additional population genomics studies of related parasites. Furthermore, deeper sequencing would allow for quantification of haplotype diversity within individual infections (Videvall et al., 2017); such diversity may also be relevant for understanding the relationship between host shifts and parasite speciation.

We have demonstrated genetic population structure of a generalist parasite among the genera of its hosts. The mechanism underlying this population structure remains unknown. Our results suggest that parasites are able to diverge and perhaps eventually speciate in sympatry, although isolation among host species in sympatry is not necessarily equivalent to sympatric speciation (Pérez-Tris et al., 2007). This result is important for considering modes of parasite speciation (Pérez-Tris et al., 2007; Ricklefs et al., 2014). Future work will need to establish the relationship between parasite population structure and speciation, the frequency with which generalist parasites show signals of genetic population structure among their host species in sympatry, and the mechanisms underpinning such genetic population structure. The field of avian haemosporidian evolutionary ecology has grown rapidly over the last 2 decades with the development of DNA barcoding of parasite lineages (Bensch et al., 2000; Hellgren et al., 2004) and the establishment of a unifying database to facilitate comparative analyses (Bensch et al., 2009). Novel genomic techniques like sequence capture hold promise for again advancing the field to allow for informative tests of novel hypotheses and a better general understanding of vector-transmitted parasite evolution.

## Supporting information

Ellis et al. supplementary material 1 Ellis et al. supplementary material

Ellis et al. supplementary material 2 Ellis et al. supplementary material

Ellis et al. supplementary material 3 Ellis et al. supplementary material

Ellis et al. supplementary material 4 Ellis et al. supplementary material

Ellis et al. supplementary material 5 Ellis et al. supplementary material

Ellis et al. supplementary material 6 Ellis et al. supplementary material

Ellis et al. supplementary material 7 Ellis et al. supplementary material

Ellis et al. supplementary material 8 Ellis et al. supplementary material

## Data Availability

All raw sequence data are available on GenBank (accession number: PRJNA1111424).

## Supplementary material

The supplementary material for this article can be found at https://doi.org/10.1017/S0031182024001641.
