# Supplementary material for: A generalist vector-transmitted parasite exhibits population genetic structure among host genera
Source: Parasitology. 2025 Jan 21;152(1):82–91. doi: 10.1017/S0031182024001641 (PMC12088918; doi:10.1017/S0031182024001641)
Supplement: Ellis et al. supplementary material 1 — Ellis et al. supplementary material [file S0031182024001641sup001.docx]

**Supplementary Table 1.** Sample information including the Sample ID, the Sample ID in the VCF file, the Bensch Lab Database ID number (Database NR), unique ring number given to the bird in the field (RingNR), the host species, date the host was sampled, and the sequence capture pool that the sample was added to.

**Supplementary Table 2.** The targeted exon regions (Target_Reference) and genes (HtGene) from the original *H. tartakovskyi* reference genome assembly and annotation and the corresponding information in the latest assembly and annotation (seqid is the reference contig, type is whether it is a protein coding gene or pseudogene, attributes associated with the gene and gene id are included; Description is copied from the attributes column). Targeted genes that do not have a corresponding gene listed are no longer annotated as genes in the latest *H. tartakovskyi* assembly.

**Supplementary Table 3.** The loadings from the first two principal components of the PCA. Variant_Position refers to the target sequence and the position in the sequence follows after the colon.

**Supplementary Table 4.** Variants with *F_ST_* > 0 when comparing infections of *Phylloscopus* and *Sylvia*. WEIR_AND_COCKERHAM_FST is the Weir and Cockerham weighted *F_ST_* value, POS is the position in the target sequence; other columns follow Supplementary Table 2.


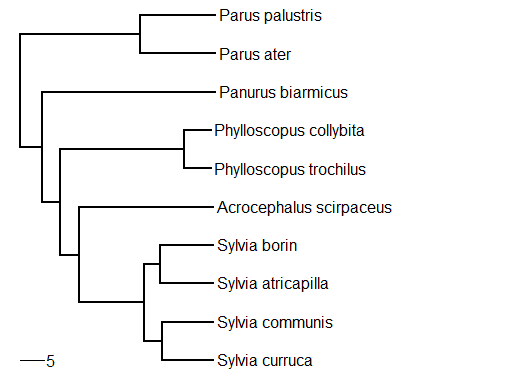


**Supplementary Figure 1.** Phylogenetic relationships among the host species in the study. Scale bar is in units of millions of years.

**
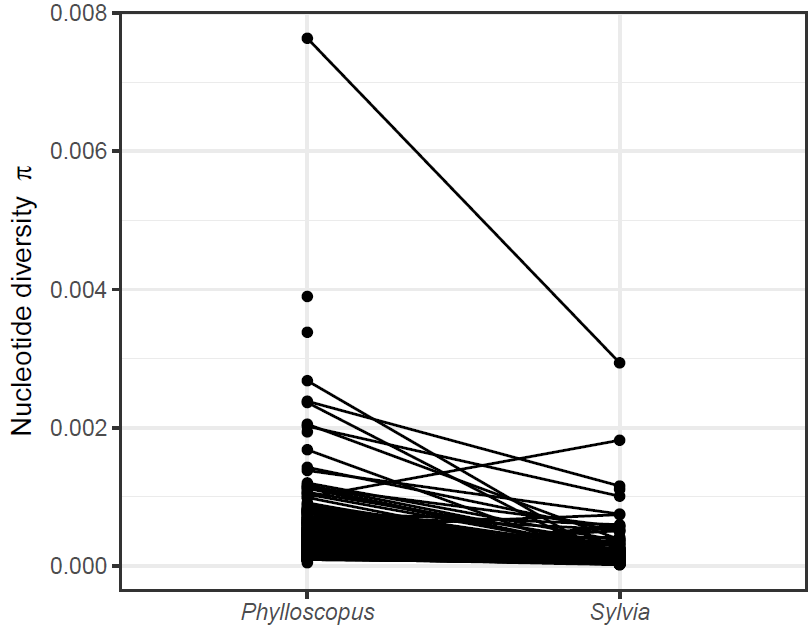
**

**Supplementary Figure 2.** Nucleotide diversity (π) calculated over 1kb windows in infections of *Phylloscopus* and *Sylvia* hosts. The same windows measured in both groups are connected with a line. Infections of *Phylloscopus* hosts had higher π than infections of *Sylvia* hosts.

**
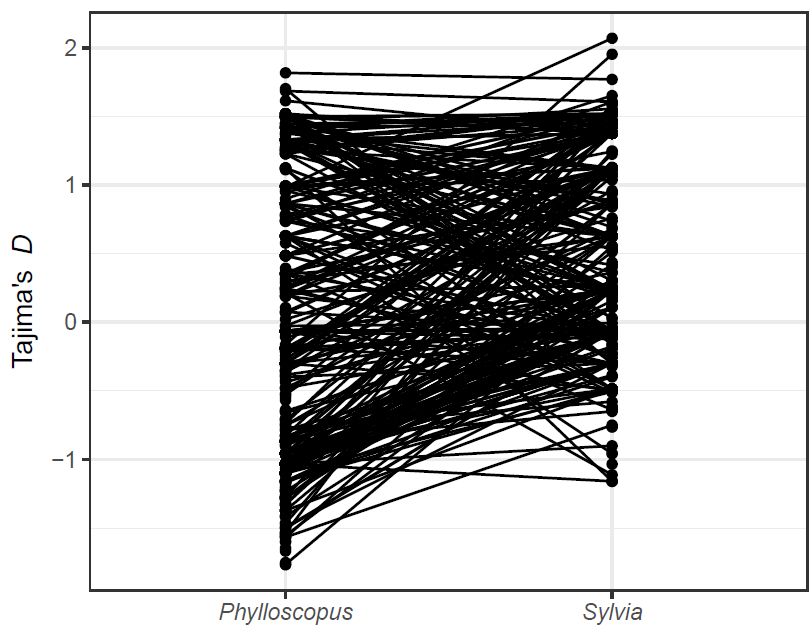
**

**Supplementary Figure 3.** Tajima’s *D* calculated over 1kb windows in infections of *Phylloscopus* and *Sylvia* hosts. The same windows measured in both groups are connected with a line. Infections of *Phylloscopus* hosts had lower *D* than infections of *Sylvia* hosts.

**Supplementary File 1.** Fasta file of targeted exons.

**Supplementary File 2.** Fasta file of probes used for sequence capture.

**Supplementary File 3.** Filtered VCF file used in the analyses.
